# Boundary work in marketized parent–school relations: an insider autoethnography of teacher emotional labor in a Chinese international school

**DOI:** 10.3389/fpsyg.2026.1877257

**Published:** 2026-08-19

**Authors:** Zhang Yue, Zhang Shikun

**Affiliations:** 1 Wycombe Abbey School Nanjing, Nanjing, Jiangsu, China; 2 School of Government, Yunnan University, Kunming, China

**Keywords:** boundary work, Chinese international school, insider autoethnography, marketized parent–school relations, professional autonomy, teacher emotional labor

## Abstract

**Background:**

The marketization of international schools embeds teachers’ work within family choice, service commitments, expectations for educational advancement, and satisfaction-based evaluation.

**Objective:**

This study examines how parent-as-client logic enters teachers’ everyday parent–school communication in a Chinese international school, how it shapes teachers’ emotional labor and perceived professional autonomy, and how teachers engage in boundary work to negotiate professional identity and occupational wellbeing.

**Methods:**

The study adopts an analytic insider/practitioner autoethnographic approach. The data comprise systematic reflective teacher logs, synthesized critical incident narratives, teacher-generated professional materials, de-identified institutional discourse materials, and reflexive analytic memos. The analysis combined thematic analysis with theory-driven coding.

**Results:**

Parent–school relations partly shifted from an educational partnership toward a client-service logic; teachers undertook invisible emotional labor in parent–school communication; parental expectations and school service commitments exerted soft pressure on professional autonomy; teachers maintained professional judgment and psychological resources through communicative, pedagogical, evidentiary, and identity boundary work; and teachers experienced identity tensions among the roles of educator, service provider, school representative, and professional decision-maker, while reconstructing professionalism through boundary work.

**Conclusion:**

This study extends research on teacher emotional labor beyond classroom interaction to marketized parent–school relations and advances research on educational marketization by foregrounding teachers’ micro-level psychological experiences. The findings suggest that boundary work is an important mechanism through which teachers sustain professional autonomy, occupational wellbeing, and professional identity. In marketized school contexts, teacher stress emerges as a psychological process jointly produced by emotional rules, organizational relations, and negotiations of professional identity.

## 1 Introduction

The expansion of international schools has reshaped the organizational and psychological conditions of teachers’ work. Under the combined influence of family choice, tuition investment, expectations for educational advancement, and school branding, teachers are expected not only to fulfil instructional responsibilities but also to respond to parents’ expectations regarding communication efficiency, individualized support, and educational outcomes. In 2024, the number of English-medium international schools worldwide exceeded 14,000, enrolling approximately 6.9 million students and generating around USD 60 billion in annual tuition revenue, with Asia accounting for a substantial proportion (ISC Research, 2024). In mainland China, parent choice, tuition investment, English-language competence, internationalized branding, expectations for progression to higher education, and individualized services jointly shape teachers’ work environment. Teachers are responsible not only for instruction and student support but are also situated within a psychological field in which school branding, service experience, and parent–school satisfaction intersect. This field alters the conditions under which teacher emotional labor and professional autonomy are enacted, and makes teachers’ roles more susceptible to redefinition through market-oriented discourse (Liu and Apple, 2024; Winchip, 2024). Educational marketization has changed the relational structure between schools and families. Within school choice and competitive mechanisms, parents may gain stronger rights to choose, evaluate, and exit, and may also be constructed as clients or consumers in school promotion, service commitments, and organizational governance (Bowe et al., 1994; Carlbaum, 2016). This study does not position parents as the source of the problem; rather, it examines how parent–school interaction becomes embedded in marketized institutional relations and how this context reshapes teachers’ emotional labor, professional autonomy, and professional identity.

Existing research on international schools and internationalized schools in China has largely focused on curriculum implementation, intercultural education, student mobility, parent choice, and teacher identity, showing that teachers in international schools often negotiate their roles amid cross-cultural mobility, organizational positioning, and market discourse (Poole and Bunnell, 2024). However, the psychological experiences of teachers in Chinese international schools remain insufficiently examined, particularly with regard to how marketized parent–school communication shapes teachers’ emotional labor, perceived professional autonomy, boundary work, professional identity, and occupational wellbeing. Accordingly, taking parent-as-client logic as its point of entry, this study adopts an analytic insider/practitioner autoethnographic approach to examine how marketized parent–school relations shape teachers’ emotional labor, professional autonomy, boundary work, and professional identity in a Chinese international school. This study addresses the following research questions:

*RQ1*. How does parent-as-client logic enter teachers’ everyday parent–school communication in a Chinese international school?

*RQ2*. How does this logic shape teachers’ emotional labor and perceived professional autonomy?

*RQ3*. How do teachers engage in boundary work to negotiate professional identity, protect professional judgment, and sustain occupational wellbeing in marketized parent–school relations?

## 2 Literature review

### 2.1 Marketized parent–school relations and parent-as-client logic

Rather than treating marketization as only a macro-level policy process, this study focuses on how marketized relations enter teachers’ everyday communication with parents. In internationalized schools, family choice, tuition investment, school branding, and expectations for educational advancement may turn parent–school communication into a space where educational partnership and service-oriented expectations overlap (Liu and Apple, 2024; Winchip, 2024; Bowe et al., 1994; Carlbaum, 2016).

In this study, parent-as-client logic refers to an organizational relation in which teachers are expected not only to teach, but also to explain, reassure, document, respond, and make professional work visible. This logic does not define parents as problematic; rather, it describes how parental expectations, school reputation, outcome accountability, and service commitments reshape the conditions of teachers’ work. This provides the relational context for examining teachers’ emotional labor, perceived professional autonomy, and boundary work.

### 2.2 Teacher emotional labor in parent–school communication

Emotional labor refers to the regulation of emotional experience and expression in accordance with workplace norms and interactional expectations (Hochschild, 2012). In teaching, emotional labor commonly involves surface acting, deep acting, and genuine emotional expression (Horner et al., 2020; Jaikla and Piyakun, 2025), and is closely connected to care, classroom order, and professional performance. In the Chinese educational context, teachers are often expected to balance passionate commitment with rational restraint (Yin and Lee, 2012).

Existing studies have shown that teachers’ emotional labor is associated with teacher efficacy, occupational commitment, wellbeing, and emotional exhaustion (Chang, 2009; Yin et al., 2017; Zheng et al., 2020; Yin et al., 2019). However, this literature has focused mainly on classroom and student interactions, with less attention to parent satisfaction, service expectations, and client-oriented communication in marketized parent–school relations (Sutton and Wheatley, 2003; Zhai and Guo, 2025). In this study, teacher emotional labor is therefore understood not only as caring interaction, but also as invisible service labor through which teachers maintain calmness, warmth, responsiveness, and professional restraint in parent–school communication.

### 2.3 Professional autonomy, teacher identity, and occupational wellbeing

Professional autonomy is an important psychological resource in teachers’ work. Teachers’ perceived autonomy is closely related to work engagement, job satisfaction, and emotional exhaustion (Skaalvik and Skaalvik, 2014). In marketized international schools, teachers’ instructional judgment may not be directly restricted by formal administrative commands, but it may be subtly shaped by parental expectations, anticipated complaints, school branding, and service commitments. Professional autonomy may therefore be experienced not only as formal decision-making authority, but also as a psychological sense of freedom within organizational relations.

This pressure also has an identity dimension. Teachers are educational professionals, but they may also be expected to act as service providers, communicators, emotional reassurance providers, and representatives of the school’s image. Teacher identity is not a stable label, but is continually formed through emotional experience, self-understanding, and organizational relations (Beauchamp and Thomas, 2009; Zembylas, 2003). When teachers repeatedly monitor their tone, justify their decisions, anticipate parent satisfaction, and adjust the presentation of teaching, professional identity and occupational wellbeing may become closely connected to the quality and boundaries of parent–school communication. Longitudinal evidence on teacher burnout further suggests that teacher exhaustion is shaped by work climate or pressure, support, conflict, organizational context, and individual characteristics (Mijakoski et al., 2022).

### 2.4 Boundary work as professional self-protection

Boundary work provides a useful concept for understanding how teachers respond to marketized parent–school relations. It refers to the effort to distinguish legitimate professional responsibility from expectations that exceed the tolerable limits of work (Gieryn, 1983). In this study, boundary work is used to analyze how teachers protect professional judgment and emotional resources without rejecting parent–school collaboration.

In parent–school communication, boundary work may appear in communicative, pedagogical, evidentiary, and identity forms. Communicative boundaries involve response time, communication channels, and the rhythm of interaction. Pedagogical boundaries redirect discussion toward curriculum goals, learning needs, and assessment standards. Evidentiary boundaries use learning evidence, written records, assessment criteria, and curriculum explanations to support professional judgment. Identity boundaries help teachers distinguish between being responsive to parents and being fully absorbed into the role of service provider. Through these forms of boundary work, teachers may sustain professional autonomy, occupational wellbeing, and professional identity in marketized school relations.

## 3 Research methods

### 3.1 Research design

This study adopts an analytic insider/practitioner autoethnographic approach (Anderson, 2006; Chang, 2008; Bullough Jr and Pinnegar, 2001). The teacher-researcher’s reflective experience serves as situated evidence for interpreting how marketized parent–school relations are lived and negotiated in everyday school work. The corpus combines reflective teacher logs, synthesized critical incident narratives, teacher-generated professional materials, institutional discourse materials, and reflexive memos, allowing lived experience to be connected with theory-informed coding, negative case analysis, and reflexive interpretation. The purpose of the study is not to reconstruct any single event, but to draw on the micro-processes of teachers’ everyday work to illuminate forms of practical experience that are often obscured by institutional texts and formal interviews, particularly those involving parent–school communication, professional judgment, emotional regulation, and identity negotiation.

### 3.2 Research site

The research site is an anonymized urban internationalized school in mainland China. The school primarily serves Chinese families and emphasizes international curricula, English-language development, individualized support, parent–school collaboration, and preparation for educational advancement. To reduce the risk of site identification, this article does not disclose information that could point to a specific institution, including the city in which the school is located, its real name, tuition level, curriculum programs, student enrollment, or university progression data.

On the one hand, the school frames educational quality, international experience, and students’ educational progression as professional commitments. On the other hand, it must also respond to families’ expectations regarding service experience, communication efficiency, individualized attention, and the visibility of outcomes. Teachers’ work therefore extends beyond classroom instruction to include curriculum explanation, learning feedback, parent–school communication, risk anticipation, emotional reassurance, and the maintenance of the school’s image. In these everyday practices, parent-as-client logic does not always appear as an explicit slogan; rather, it enters teachers’ professional lives through communicative tone, feedback frequency, explanatory obligations, anticipated complaints, and pressure related to satisfaction.

### 3.3 Researcher positionality and reflexivity

The researcher previously taught for 4 years in Chinese international schools, undertaking classroom instruction, parent–school communication, pedagogical explanation, and student support. This insider position enabled access to aspects of teachers’ everyday experience that are difficult for external researchers to observe, such as deliberating over wording before replying to parents, anticipating potential complaints, shifting between professional and service-oriented language, and reflecting on teacher identity after emotional fatigue. At the same time, the researcher was both the experiencing subject and the interpreter of the data, which could have led to an overemphasis on personal stress, insufficient attention to the constructive dimensions of parent–school collaboration, or a direct translation of personal emotion into organizational critique. To reduce positional bias, the study involved the continuous writing of reflexive memos to document emotional responses, interpretive assumptions, and possible biases. During coding, the researcher actively searched for negative cases and cross-referenced reflective teacher logs, synthesized critical incidents, teacher-generated professional materials, and institutional discourse materials to avoid making claims on the basis of a single strand of experience. The researcher’s positionality was therefore treated not as an interference to be eliminated, but as an analytic entry point for understanding teachers’ emotional labor, boundary work, and the negotiation of professional autonomy (Appendix 1). Reflective excerpts and synthesized vignettes were used as analytic anchors connecting lived teacher experience with broader organizational relations.

### 3.4 Data sources and composition

The formal data for this study were drawn primarily from a systematic reflection cycle lasting 16 weeks. The researcher’s 4 years of work experience in international schools served as background for understanding the field, identifying contextual patterns, and developing analytic sensitivity, but it was not treated as equivalent to the full dataset. The data comprised reflective teacher logs, synthesized critical incident narratives, teacher-generated professional materials, public or de-identified institutional discourse materials, and reflexive analytic memos. All materials focused on parent–school communication, parental expectations, instructional decision-making, emotional regulation, professional autonomy, boundary management, and tensions in teacher identity.

### 3.5 Data collection

Data collection proceeded in three stages. The first stage involved retrospective mapping of the field. Drawing on 4 years of teaching experience in international schools, the researcher conceptually organized recurring situations related to parent–school communication, service expectations, professional judgment, and emotional labor. This stage was used to clarify the observational focus for the subsequent period of systematic reflection, rather than to incorporate past experience in its entirety into the analysis.

The second stage involved the systematic writing of reflective teacher logs. Over 16 weeks, the researcher wrote regularly around parent satisfaction, parent–school communication, emotional regulation, professional autonomy, boundary management, and occupational wellbeing, with particular attention to the judgment processes involved in pedagogical explanation, communicative response, emotional control, self-doubt, and boundary setting. The logs did not record identifiable information about students, parents, colleagues, or administrators, nor were they intended to reconstruct specific events.

The third stage involved the organization of materials and the writing of analytic memos. The researcher categorized teacher-generated materials such as curriculum explanations, assessment interpretations, feedback templates, and drafts prepared for communication, and wrote synthesized critical incident narratives based on recurring patterns in the logs. Public or de-identified institutional discourse materials were also collected as contextual references. Throughout this process, the researcher continued to write reflexive memos documenting theme development, interpretive uncertainties, possible biases, and negative cases.

### 3.6 Data analysis

This study combined thematic analysis with theory-driven coding. First, the researcher repeatedly read the reflective teacher logs, synthesized critical incidents, teacher-generated professional materials, institutional discourse materials, and reflexive memos to develop a holistic understanding of the relationships among the marketized school context, the pressures of parent–school interaction, and teachers’ emotional labor. Open coding was then conducted to identify initial codes from the data, including pressure related to parent satisfaction, anticipated complaints, service-oriented tone, emotional suppression, professional self-doubt, defensive documentation, boundary setting, and identity tension.

On this basis, the researcher compared the initial codes with theoretical concepts related to emotional labor, professional autonomy, educational marketization, service labor, teacher identity, and boundary work, thereby developing theory-informed codes. Through multiple rounds of comparison, the analysis generated core themes, including client-service logic, invisible emotional labor, soft pressure on professional autonomy, boundary work, identity tension, and the reconstruction of occupational wellbeing. After coding was completed, Python was used to organize the coding matrix and conduct descriptive statistics on coding frequency, thematic distribution, comparisons across data types, and co-occurrence relationships, which served to support thematic interpretation. Negative case analysis was also conducted to distinguish constructive parent–school communication from service-oriented pressure, thereby avoiding the reduction of complex parent–school relations to a simple opposition. Coding was used to trace recurrent patterns across the autoethnographic corpus, while interpretation returned to reflective excerpts and synthesized incidents to preserve the experiential sequence of teacher sense-making.

### 3.7 Research trustworthiness

The trustworthiness of the study was strengthened through thick description, data triangulation, reflexive documentation, negative case analysis, and an audit trail. Thick description was reflected in the detailed presentation of the marketized context of international schooling, parent–school communication scenarios, teachers’ emotional regulation, and processes of professional judgment, with particular attention to how pressure entered teachers’ work through communication frequency, language expectations, explanatory obligations, brand commitments, and anticipated complaints. Data triangulation was achieved by cross-referencing multiple types of material: reflective logs captured teachers’ immediate experience; synthesized critical incidents revealed recurring situational patterns; teacher-generated professional materials demonstrated how professional language was organized; institutional discourse materials provided contextual insight into school culture; and reflexive memos documented the interpretive process. At the same time, the researcher continuously examined the interpretive biases that might arise from their insider position and actively analyzed negative cases to preserve the complexity of parent–school communication. Coding records, the process of theme revision, and analytic memos together formed an audit trail, making the interpretive process traceable.

### 3.8 Ethical statement

This study did not recruit human participants, conduct interviews, administer questionnaires, record classroom audio or video, or observe parent meetings. Nor did it analyze identifiable data from students, parents, colleagues, or administrators. The research materials were mainly derived from the researcher’s own reflective logs, teacher-generated professional materials, analytic memos, and public or de-identified institutional discourse materials. Critical incidents were presented as synthesized narratives and do not correspond to any single real person, family, class, colleague, or school event. Their function is to represent recurring structural tensions rather than to reconstruct individual cases. All information relating to schools, persons, grade levels, curricula, and organizational arrangements was anonymized, blurred, and de-identified. Given this research design, the study did not involve identifiable personal data or the recruitment of human participants; therefore, ethical approval was not required.

## 4 Results

The findings move between reflective episodes and corpus-level patterns. Reflective logs and synthesized incidents provide experiential anchors, while coding patterns indicate how these experiences recurred across the wider autoethnographic corpus.

### 4.1 From educational partnership to client-service logic

This study analyzed 90 autoethnographic materials, including 44 reflective teacher logs, 16 teacher-generated professional materials, 12 reflexive analytic memos, 10 synthesized critical incident narratives, and 8 public or de-identified institutional discourse materials. These materials captured teachers’ everyday reflections, the organization of professional texts, interpretive processes, typical interaction patterns, and marketized school discourse, thereby providing complementary evidence for the thematic analysis (Table 1). Across the corpus, this service-oriented shift was reflected in codes such as “parent-as-client logic,” “emotional reassurance work,” “service visibility,” “expectations of immediate response,” “outcome accountability,” and “school brand consistency” (Table 2).

**Table 1:** Composition and analytic uses of the autoethnographic corpus.

Data type	*n*	Percentage (%)	Analytic use
Reflective teacher logs	44	48.9	To identify everyday manifestations of teacher emotional labor, pressure on professional autonomy, and boundary management
Teacher-generated professional materials	16	17.8	To examine how teachers organize professional language, explain pedagogical decisions, and maintain professional boundaries
Reflexive analytic memos	12	13.3	To trace the interpretive process and reduce single-perspective bias in insider research
Synthesized critical incident narratives	10	11.1	To represent structural tensions and typical interaction patterns in marketized parent–school relations
Public or de-identified institutional discourse materials	8	8.9	To provide contextual evidence of school discourse and organizational expectations
Total	90	100	—

**Table 2:** Research questions, result themes, and coding framework.

Research question	Corresponding result theme	Key codes	Analytic focus
RQ1. How does parent-as-client logic enter teachers’ everyday parent–school communication in a Chinese international school?	R1 From educational partnership to client-service logic: the service-oriented shift in parent–school relations	C01 parent-as-client logic; C02 expectations of immediate response; C03 emotional reassurance work; C04 service visibility; C05 outcome accountability; C06 school brand consistency	To examine how parent-as-client logic enters everyday parent–school communication through response expectations, service visibility, outcome accountability, and school brand consistency
RQ2. How does this logic shape teachers’ emotional labor and perceived professional autonomy?	R2 Emotional labor as invisible service labor	C07 surface acting; C08 deep acting; C09 emotional containment; C10 professionally bounded warmth	To examine how teachers regulate emotions in parent–school communication while maintaining a professional and service-oriented communicative stance
RQ2. How does this logic shape teachers’ emotional labor and perceived professional autonomy?	R3 Professional autonomy under soft pressure	C11 anticipatory adjustment; C12 defensive documentation; C13 adjustment of instructional presentation; C14 negotiation of professional autonomy	To examine how parental expectations and school service commitments create soft pressure on teachers’ professional autonomy through anticipation, documentation, and adjustment of instructional presentation
RQ3. How do teachers engage in boundary work to negotiate professional identity, protect professional judgment, and sustain occupational wellbeing in marketized parent–school relations?	R4 Boundary work as a strategy for protecting professionalism	C15 communicative boundaries; C16 pedagogical boundaries; C17 evidence boundaries; C18 identity boundaries	To examine how teachers protect professional judgment through communicative, pedagogical, evidentiary, and identity boundaries
RQ3. How do teachers engage in boundary work to negotiate professional identity, protect professional judgment, and sustain occupational wellbeing in marketized parent–school relations?	R5 Identity tension and teacher occupational wellbeing	C19 emotional exhaustion; C20 professional self-doubt; C21 self-monitoring; C22 educator/service-provider tension; C23 professional/school-brand representative tension; C24 caregiver/exhausted-worker tension; C25 reconstruction of occupational wellbeing	To examine how teachers experience identity tension and occupational strain, and how negative cases refine the interpretation of parent–school communication beyond a one-directional service-pressure account

RQ = research question. R1–R5 refer to the five result themes generated from thematic analysis. Table 2 lists the substantive codes used to interpret each result theme. Corpus-level descriptive summaries and visualizations retained the original codebook links: R1 = C01–C06; R2 = C07–C10; R3 = C11–C14; R4 = C15–C18, C26, and C29; and R5 = C19–C25, C27, and C28. C26 was used as a cross-cutting negative-case code. C27–C29 served reflexive and ethical documentation functions and were not interpreted as standalone substantive findings.

In one reflective log, a parent’s reference to high tuition and service quality shifted the teacher’s response from pedagogical explanation to service management. The teacher redirected the conversation to learning support, assessment criteria, and a follow-up plan, while feeling that the professional issue had been pulled into a consumer frame. The entry illustrates how client-service logic entered everyday communication without necessarily appearing as an explicit complaint.

The reflective logs and synthesized incidents suggest that “parent-as-client” is not a single label, but a relational position jointly shaped by tuition investment, expectations for educational advancement, individualized support, timely response, and school branding. Teachers are expected not only to deliver instruction and feedback, but also to ensure that instructional support is visible, intelligible, and recognized by parents. Parents are therefore positioned both as educational partners and as recipients of service who are attentive to outcomes, while teachers, beyond their instructional role, assume additional responsibilities for explaining, responding, and demonstrating professional value.

In one synthesized incident, a teacher received several non-urgent parent messages in the evening and felt pressure to respond immediately, fearing that a delay might be seen as indifference. The teacher instead replied in writing during working hours the next day and moved the more complex issue to a formal meeting. This example shows how client-service logic entered teachers’ work through expectations of promptness, reassurance, and constant communicative availability rather than through direct demands. Additional synthesized and de-identified critical incident narratives are provided in Appendix 2.

### 4.2 Emotional labor as invisible service labor

**Figure 1:**
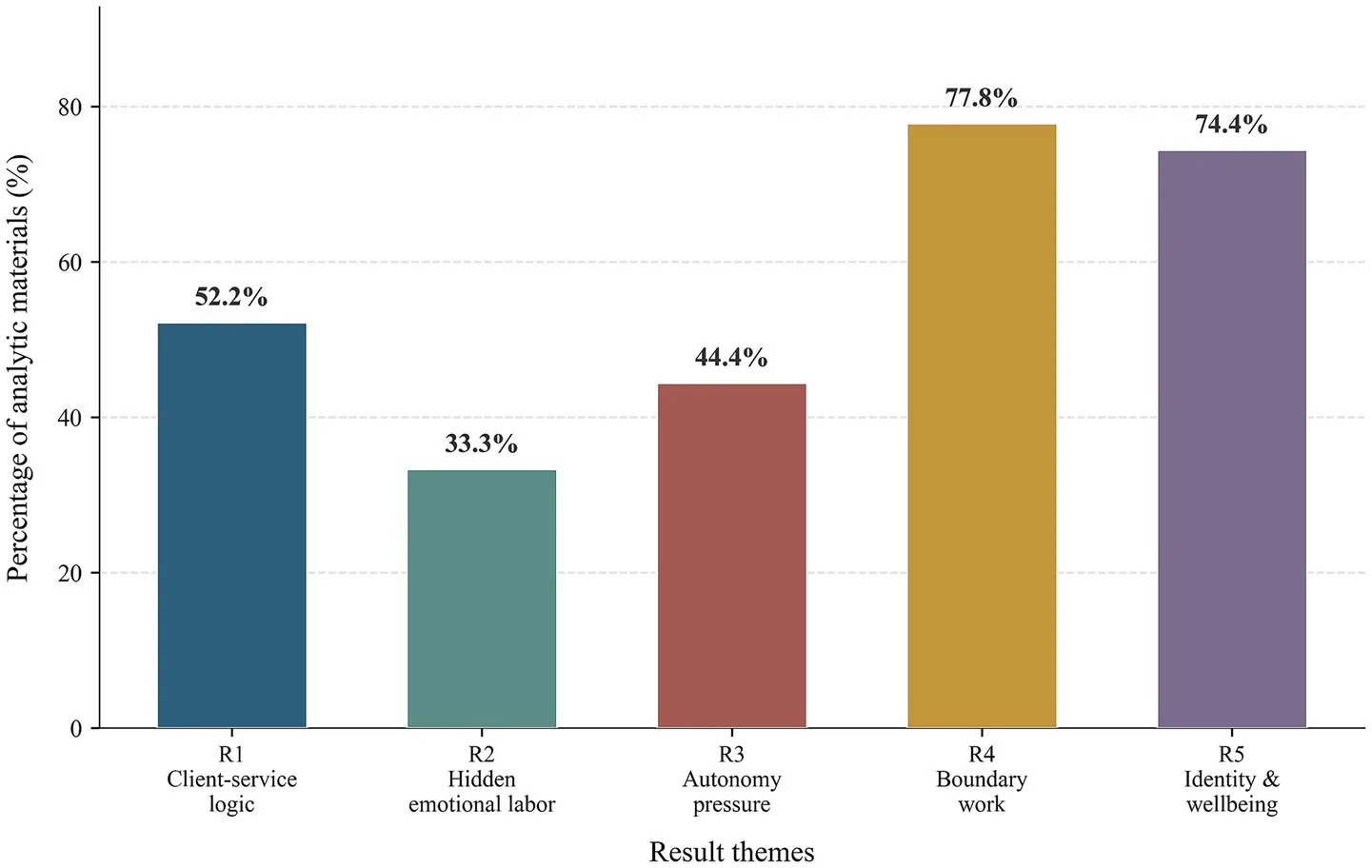
Coverage of the five core themes across the autoethnographic corpus.

At the corpus level, the distribution of themes provided further support for this pattern: boundary work and identity tension had the broadest coverage, while emotional labor appeared more selectively around specific parent–school interactions (Table 3; Figure 1). This indicates that emotional labor does not always appear as an explicit task, but is embedded in teachers’ everyday parent–school communication. Across the reflective logs and synthesized incidents, teachers’ emotional labor appeared as surface acting, deep acting, emotional containment, and professionally bounded warmth. Even when fatigued, tense, or questioned, teachers were still expected to remain patient and stable. At the same time, they attempted to reinterpret parents’ anxiety, dissatisfaction, or questioning as issues of information asymmetry or educational expectations. In communication, teachers also had to absorb and buffer external emotions while maintaining communicative order and a professional stance in a gentle but bounded manner. Emotional labor, therefore, is not simply “patient communication”; rather, it is an ongoing form of emotional regulation undertaken by teachers between service-oriented expectations and professional judgment.

**Table 3:** Theme-level descriptive coding summary.

Theme	Documents with theme	Percentage (%)	Total code hits	Mean hits per document
R1 Client-service logic in parent–school relations	47	52.2	75	0.83
R2 Emotional labor as invisible service labor	30	33.3	46	0.51
R3 Professional autonomy under soft pressure	40	44.4	58	0.64
R4 Boundary work and the protection of professionalism	70	77.8	104	1.16
R5 Identity tension and occupational wellbeing	67	74.4	132	1.47

Values are based on document-level descriptive coding. Percentages refer to the proportion of analytic materials, not participants.

A second log captured the emotional regulation involved in parent–school communication: “I had to push down my irritation and respond to the parent’s anxiety in a steady tone.” In another entry, the teacher-researcher wrote, “I tried to understand the questioning as anxiety about the child’s frustration rather than as a denial of my teaching.” These accounts show how emotional containment and deep acting were used to keep communication stable while preserving a professional stance.

Representative excerpts illustrating surface acting, deep acting, emotional containment, and professionally bounded warmth are provided in Appendix 3. These excerpts show how emotion regulation appeared in concrete acts of maintaining tone, reframing parental questioning, containing irritation, and responding warmly while preserving professional limits.

### 4.3 Professional autonomy under soft pressure

**Figure 2:**
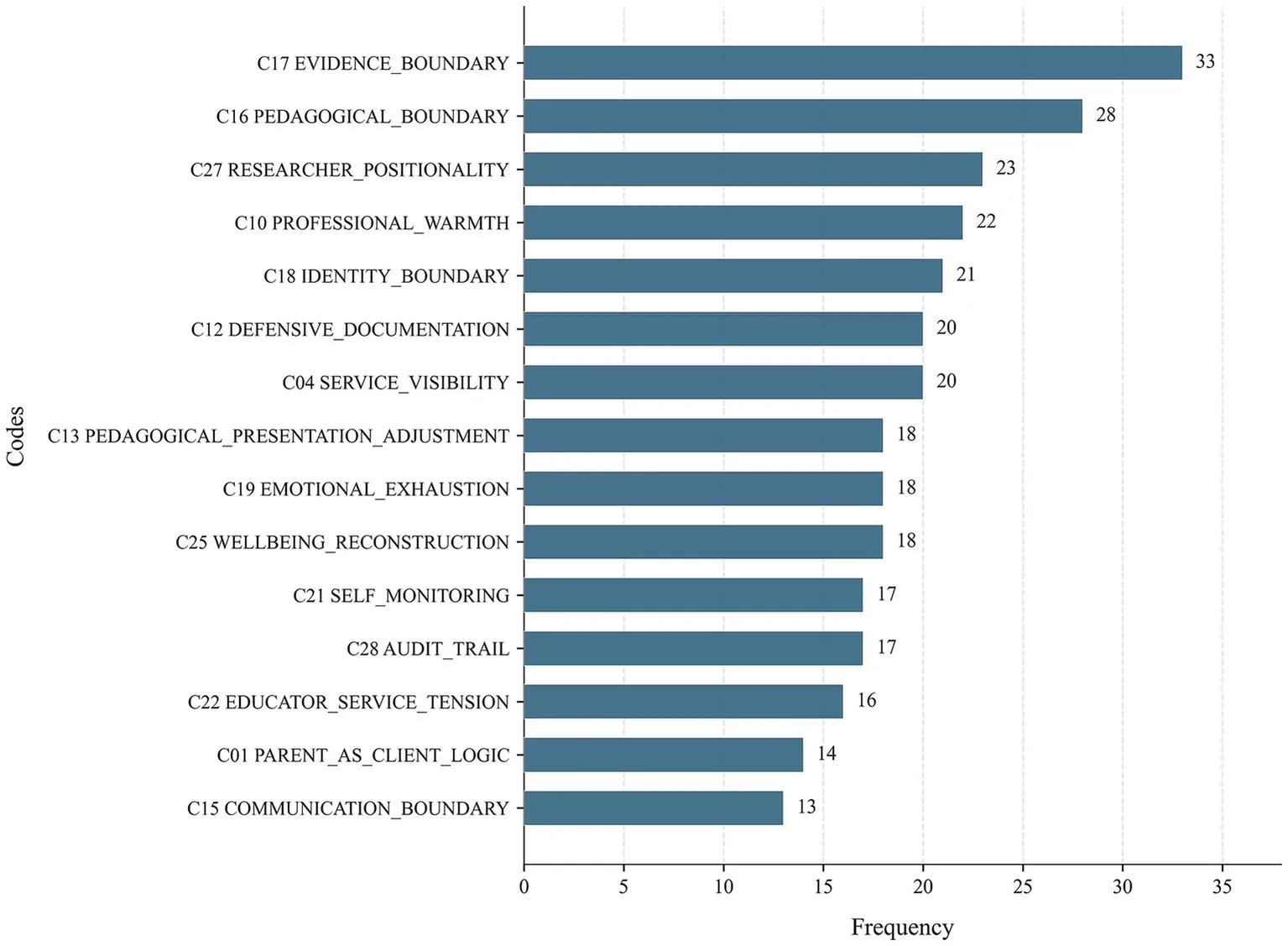
Frequency distribution of high-frequency codes in the full coding matrix.

Soft pressure on professional autonomy often appeared before direct conflict occurred. When preparing for a parent meeting, the teacher-researcher wrote: “I kept revising the meeting outline. I was not only preparing content, but also preparing emotion and tone.” This anticipatory pressure was also visible in high-frequency codes related to “defensive documentation,” “adjustment of instructional presentation,” and “negotiation of professional autonomy” (Table 4; Figure 2). “Anticipatory adjustment” in the materials referred to teachers’ consideration, before making instructional decisions, of whether parents would understand those decisions, whether they might raise questions, and whether additional explanation would be needed. “Defensive documentation” referred to teachers’ use of more detailed homework instructions, assessment criteria, and feedback records to support professional judgment. “Adjustment of instructional presentation” did not necessarily mean that instructional goals had changed; rather, it referred to teachers’ adjustment of how learning outcomes, feedback processes, and classroom arrangements were expressed so that they could be more readily seen and understood by parents. Thus, professional autonomy did not appear in the materials as a binary condition of presence or absence. Teachers still retained instructional judgment, but that judgment had to be continuously explained, documented, and presented in order to maintain balance among parental expectations, school requirements, and professional principles. Professional autonomy was therefore enacted as a state of ongoing negotiation.

**Table 4:** High-frequency codes in the full coding matrix.

Code	Code label	Linked theme	Freq.	% of docs
C17	Evidence boundaries	R4 Boundary work and protection of professionalism	33	36.7
C16	Pedagogical boundaries	R4 Boundary work and protection of professionalism	28	31.1
C27	Reflection on researcher positionality	R5 Identity tension and occupational wellbeing	23	25.6
C10	Professionally bounded warmth	R2 Emotional labor as invisible service labor	22	24.4
C18	Identity boundaries	R4 Boundary work and protection of professionalism	21	23.3
C04	Service visibility	R1 From educational partnership to client-service logic	20	22.2
C12	Defensive documentation	R3 Professional autonomy under soft pressure	20	22.2
C25	Reconstruction of occupational wellbeing	R5 Identity tension and occupational wellbeing	18	20
C19	Emotional exhaustion	R5 Identity tension and occupational wellbeing	18	20
C13	Adjustment of instructional presentation	R3 Professional autonomy under soft pressure	18	20
C28	Audit trail	R5 Identity tension and occupational wellbeing	17	18.9
C21	Self-monitoring	R5 Identity tension and occupational wellbeing	17	18.9
C22	Educator/service-provider tension	R5 Identity tension and occupational wellbeing	16	17.8
C01	Parent-as-client logic	R1 From educational partnership to client-service logic	14	15.6
C15	Communicative boundaries	R4 Boundary work and protection of professionalism	13	14.4

Frequency refers to the number of analytic materials in which the code appeared.

Additional examples of anticipatory adjustment, defensive documentation, instructional presentation adjustment, and evidence boundary are provided in Appendix 3. One teacher-generated communication draft, for example, reorganized a parent explanation around learning objectives, assessment criteria, and follow-up evidence, showing how professional materials were used to make pedagogical judgment visible without surrendering that judgment to satisfaction-based evaluation.

### 4.4 Boundary work as a strategy for protecting professionalism

**Figure 3:**
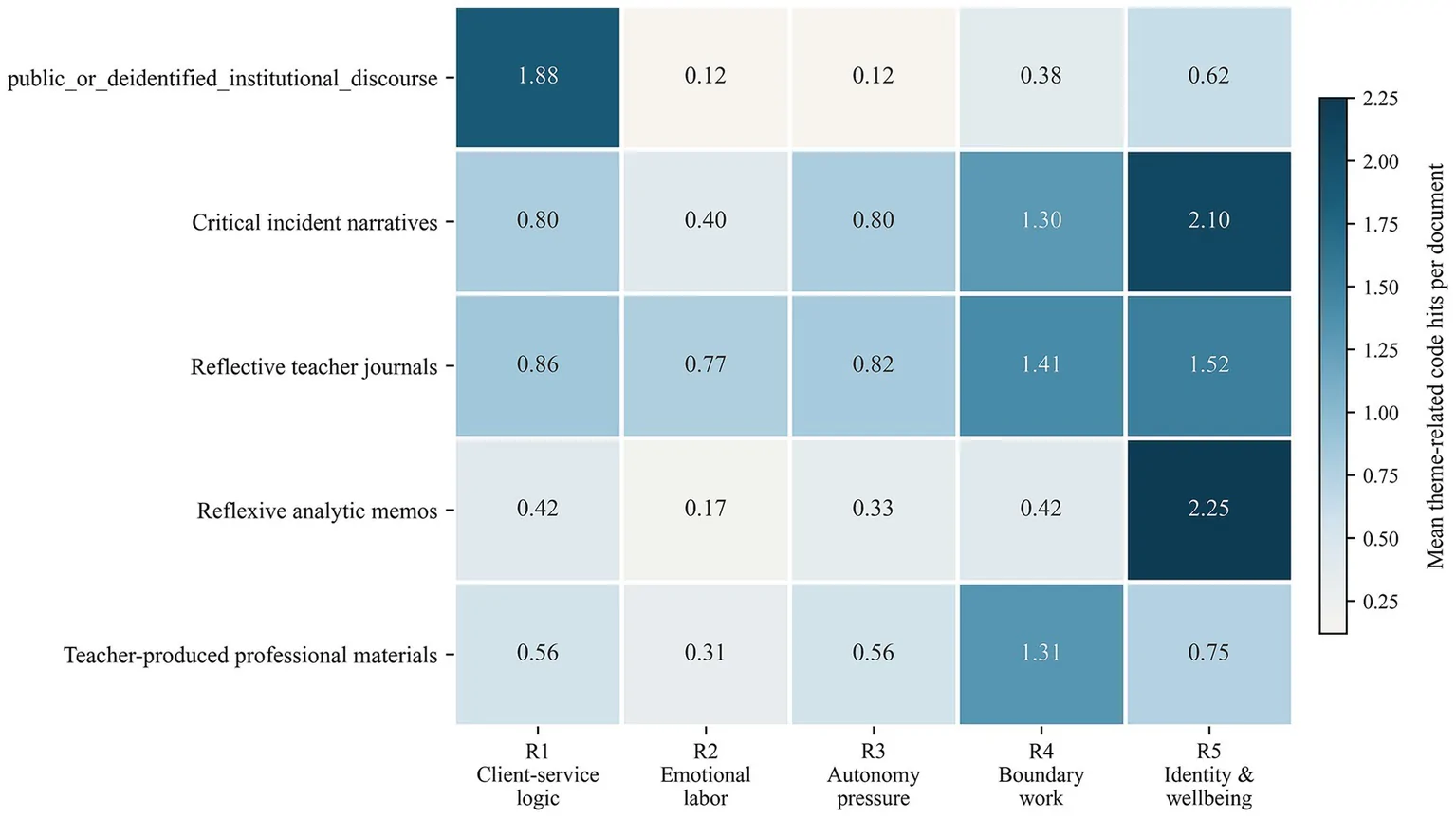
Distribution of result themes across data types.

Boundary work became visible when the teacher-researcher began to separate general learning issues from urgent safety concerns. General learning-related messages were answered during fixed working-hour periods, whereas urgent safety issues were treated separately. This distinction did not reduce communication with parents; it helped keep communication educational, accurate, and emotionally sustainable. In other words, boundary work was both an outcome of teachers’ reflection on stressful experiences and a practice embedded in homework instructions, assessment criteria, communication preparation, and typical interaction patterns (Table 5; Figure 3). Boundary work in the materials mainly included four types. Communicative boundaries involved the management of response time, communication channels, and communicative rhythm, so as to prevent parent–school communication from extending indefinitely into teachers’ private time and emotional space. Pedagogical boundaries were reflected in teachers’ efforts to redirect discussions back to students’ learning needs, curriculum goals, and assessment standards. Evidence boundaries involved teachers’ use of curriculum explanations, homework requirements, learning objectives, and feedback records to support professional judgment. Identity boundaries were reflected in teachers’ redrawing of the line between “service provider” and “educational professional.” These patterns suggest that teachers did not protect professionalism by reducing communication, but by reorganizing communication through boundary work. Boundary work was not a rejection of parents, but a practical means of sustaining professional judgment, emotional resources, and occupational identity within complex communicative relations.

**Table 5:** Distribution of result themes across data types.

Data type	*n*	R1 Client-service logic	R2 Emotional labor	R3 Autonomy pressure	R4 Boundary work	R5 Identity and wellbeing
Reflective teacher logs	44	0.86	0.77	0.82	1.41	1.52
Teacher-generated professional materials	16	0.56	0.31	0.56	1.31	0.75
Reflexive analytic memos	12	0.42	0.17	0.33	0.42	2.25
Synthesized critical incident narratives	10	0.8	0.4	0.8	1.3	2.1
Public or de-identified institutional discourse materials	8	1.88	0.12	0.12	0.38	0.62

Values indicate the mean number of theme-related code hits per document within each data type.

### 4.5 Identity tension, occupational wellbeing, and the reconstruction of teacher professionalism

**Figure 4:**
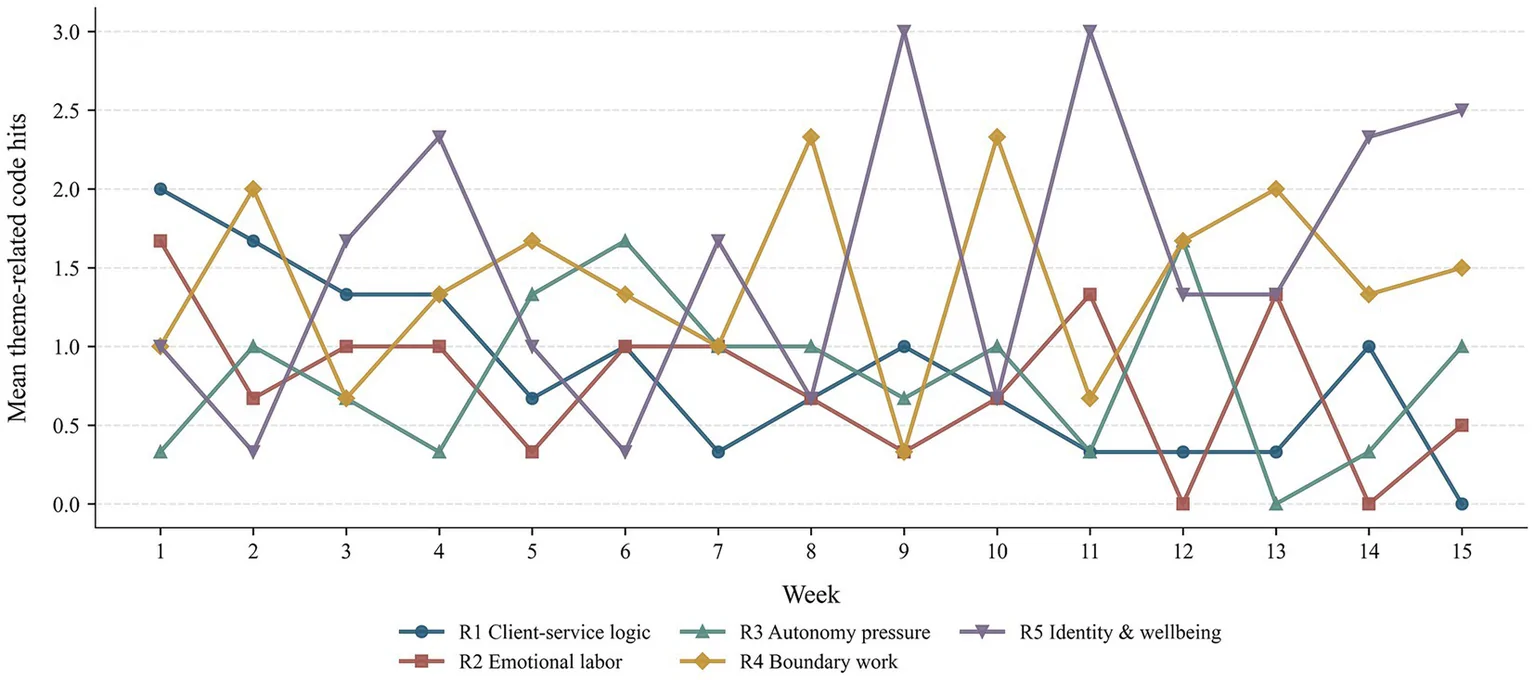
Weekly trajectory of core themes in reflective teacher logs.

“Care was not false, and exhaustion was not unprofessional.” This reflection helped interpret the broader trajectory of identity tension and occupational wellbeing across the reflective logs (Figure 4). The reflective materials suggest that teachers simultaneously occupied multiple positions in everyday work, including educator, service provider, representative of the school brand, and provider of emotional reassurance. As educators, teachers needed to uphold curriculum goals and students’ long-term development. As service providers, they needed to respond to parental expectations and explain instructional arrangements. As representatives of the school brand, they also had to attend to the relationship between their own expression and the school’s image. These roles were interwoven in parent–school communication and accompanied by experiences of self-monitoring, professional self-doubt, and emotional exhaustion.

Negative case analysis further refined this interpretation. Some materials showed that parent–school communication did not always constitute service-oriented pressure; it could also promote improvements in pedagogical explanation and enhance professional transparency (Table 6). Occupational wellbeing, therefore, did not show a unidirectional decline. On the one hand, teachers experienced emotional depletion and identity strain; on the other hand, they also reinterpreted professionalism through reflective writing, evidence-based communication, and boundary setting. Teacher professionalism was thus reflected not only in classroom instructional competence, but also in the capacity to explain teaching, manage emotions, establish boundaries, and protect students’ learning interests.

**Table 6:** Negative case analysis and interpretive refinement.

Data type	Negative cases	Representative document IDs	How the negative cases refined the interpretation
Reflective teacher logs	3	RL13; RL29; RL42	Helped distinguish constructive parent–school communication from service-oriented pressure
Reflexive analytic memos	2	RM04; RM06	Supported examination of interpretive bias arising from the researcher’s insider position
Synthesized critical incident narratives	1	CI06	Prevented all interactions from being interpreted as one-directional marketized pressure
Teacher-generated professional materials	1	PM12	Indicated that professional materials may both respond to service expectations and enhance pedagogical transparency

Negative cases were used to avoid a one-directional interpretation of parent–school communication as purely marketized pressure.

Another reflective entry linked identity tension with occupational wellbeing. Through reflective writing, the teacher came to see emotional depletion not simply as a personal weakness, but as part of the structural pressure produced by parent–school relations. A negative-case log also recorded that one parent’s feedback “was not pressure, but helped me see a gap in the task explanation,” suggesting that parent expectations could also support pedagogical clarity.

### 4.6 Summary

**Figure 5:**
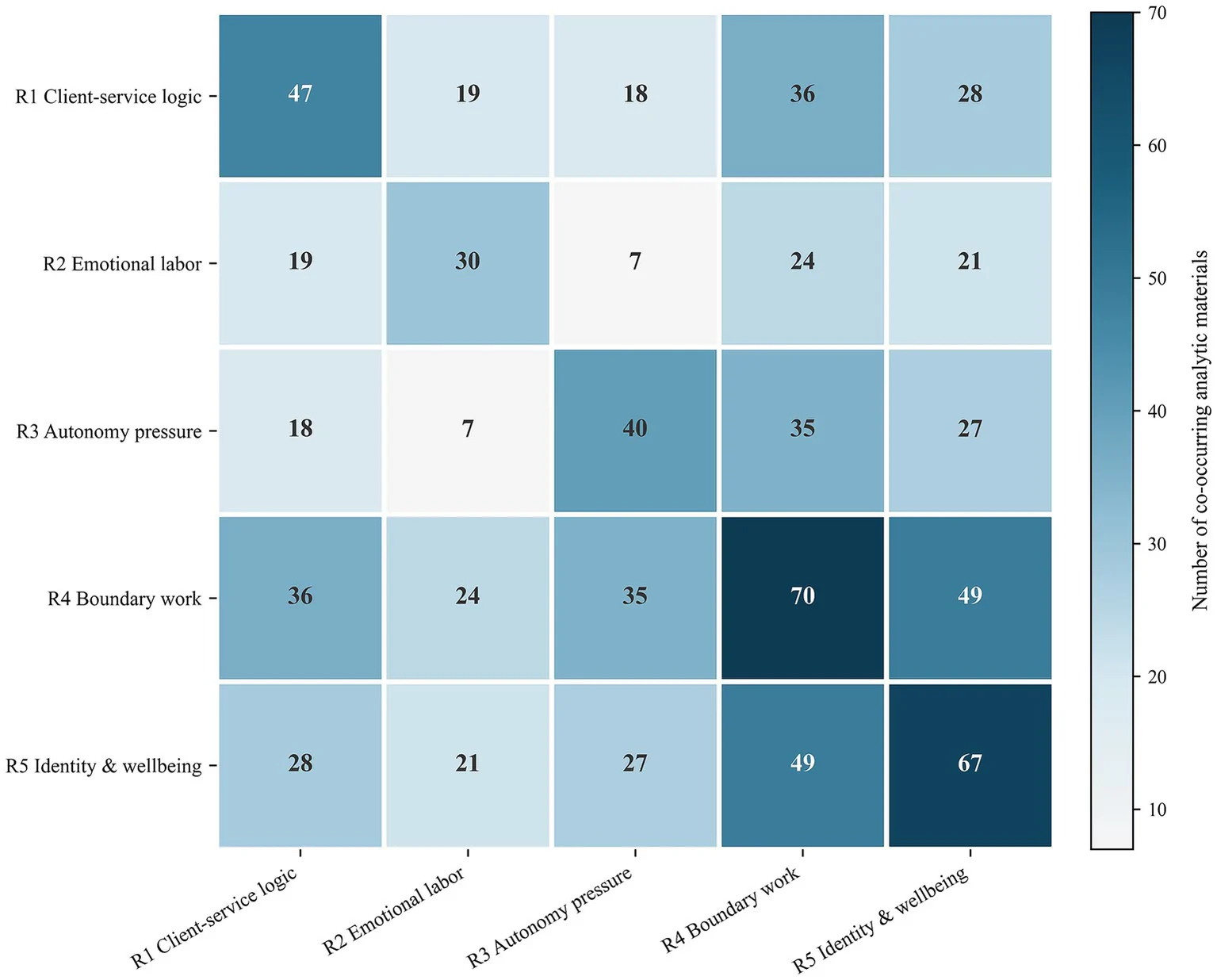
Co-occurrence relationships among the five core themes.

The co-occurrence relationships among the five themes further reveal the overall pathway of the findings. The co-occurrence between boundary work and identity tension and occupational wellbeing was the most prominent, while strong co-occurrences were also found between the service-oriented shift in parent–school relations and boundary work, and between pressure on professional autonomy and boundary work. This indicates that boundary work was located at the intersection of multiple themes, linking service-oriented parent–school relations, pressure on professional autonomy, and the reconstruction of teacher identity (Figure 5).

Taken together, the materials present a relatively clear processual chain: marketized school discourse first shapes the interactional expectation of “parents as clients”; this expectation further produces rules of emotional display in teachers’ parent–school communication; teachers consequently undertake surface acting, deep acting, emotional containment, and professionally bounded warmth; service-oriented expectations influence professional autonomy through anticipatory adjustment, defensive documentation, and adjustment of instructional presentation; and teachers maintain professional judgment through communicative, pedagogical, evidentiary, and identity boundaries. Ultimately, teachers experience identity tension among the roles of educator, service provider, school representative, and provider of emotional reassurance, while reconstructing professional identity and occupational wellbeing through sustained reflection and boundary work.

## 5 Discussion

This study found that, within the marketized Chinese international school context examined here, “parent-as-client” was not an explicit policy but an organizational logic embedded in everyday work. It entered parent–school relations through school branding, tuition investment, expectations for educational advancement, individualized service, and mechanisms of timely feedback, requiring teachers to continuously undertake work beyond instruction, including explanation, reassurance, response, and the maintenance of the school’s image. The resulting emotional labor was not merely polite communication, but an invisible form of service labor involving surface control, deep regulation, emotional containment, and professionally bounded warmth. More importantly, this logic did not directly eliminate teachers’ professional autonomy, but created soft constraints through anticipatory adjustment, defensive documentation, and communicative pressure. Teachers were not passive recipients of these pressures; rather, they maintained professional judgment through communicative, pedagogical, evidentiary, and identity boundaries, while reinterpreting their professional identity across the roles of educator, service provider, and school representative.

### 5.1 Marketized parent–school relations and rules of emotional expression

In this article, “parent-as-client” is not a moral judgment of parents, but an analytical description of organizational relations in marketized schooling. Through school choice, tuition payment, and service commitments, parents are positioned as choosers, evaluators, and recipients of service. This may change how schools present educational quality and require teachers’ professionalism to be continuously explained, validated, and negotiated in parent–school interaction (Breidenstein et al., 2023). Such negotiation does not occur only at the level of management systems, but also in every instance in which teachers reply, explain, clarify, and reassure. This finding extends the contextual boundaries of research on teachers’ emotional labor. Existing studies have tended to focus on emotional expression in classroom and student interactions, whereas this study shows that marketized parent–school communication itself can also generate rules of emotional display. When facing parents, teachers are expected to remain patient, positive, stable, and responsive, even when their internal experiences may involve fatigue, hesitation, or frustration after being questioned. Emotional display rules in school contexts are not merely a matter of personal disposition; rather, they are jointly shaped by organizational expectations and interactional norms (Diefendorff and Richard, 2003).

When parent–school communication is incorporated into the framework of service experience and brand maintenance, teachers’ emotion management shifts from an individual communicative skill to a form of organizational labor (Grandey, 2000). Digitalized and high-frequency parent–school communication further intensifies these rules. Parent messages, feedback records, and explanatory texts make it more difficult for teachers to naturally close the boundaries of work, while also increasing their self-expectations regarding response speed, warmth of wording, and emotional stability. Digital communication can enhance informational transparency, but it may also continuously intrude into teachers’ psychological space (Kuusimäki et al., 2019). The influence of client logic, therefore, does not arise only in moments of conflict; it is also present in teachers’ anticipation of potential dissatisfaction, refinement of language, and continuous maintenance of the school’s image.

### 5.2 Emotional labor, pressure on professional autonomy, and psychological costs

Teachers’ emotional labor in marketized parent–school communication is often accompanied by deeper psychological regulation. Teachers are not only expected to “speak appropriately,” but also to suppress immediate reactions, understand parental anxiety, anticipate the risk of complaints, and shift their mode of expression between professional judgment and parental acceptability. Prolonged surface acting and emotional suppression may contribute to exhaustion, detachment, and occupational burnout, which is consistent with findings in research on teacher emotional labor (Yin et al., 2019; Grandey, 2003).

This study further shows that pressure does not arise only from negative interactions themselves, but from the continuous anticipation of external evaluation. Before making instructional decisions, teachers may already begin to consider whether parents will understand, whether additional explanation will be needed, whether satisfaction may be affected, and even whether the issue may develop into a complaint. Professional autonomy, therefore, is no longer simply a matter of whether teachers formally possess decision-making authority, but becomes the psychological degree of freedom that teachers perceive within organizational relations. The Job Demands–Resources (JD-R) model suggests that sustained job demands deplete individual resources, whereas resource support helps maintain engagement and reduce exhaustion (Bakker and Demerouti, 2007). In this context, pressure related to parent satisfaction and school service commitments constitutes an implicit job demand, while a sense of professional autonomy becomes an important psychological resource through which teachers resist exhaustion.

Deep emotional labor is ambivalent in this context. By reinterpreting parental anxiety and viewing communication as part of educational support, teachers may sustain a sense of occupational meaning. However, when such self-persuasion is used over time to absorb unbounded expectations, it may also intensify psychological burden. Different combinations of emotional labor may have different implications for wellbeing, and deep acting is not inherently protective (Hülsheger and Schewe, 2011). When deep regulation is accompanied by clear boundaries, it may support professional identity; when it serves open-ended pressure for satisfaction, it may become a form of self-depletion. The key feature of soft pressure on autonomy is that it rarely appears as a direct command. More often, teachers adapt in advance to external expectations through defensive documentation, anticipatory communication, explanatory obligations, and adjustment of instructional presentation. School-based stressors are closely associated with teacher burnout (Skaalvik and Skaalvik, 2011), while autonomy support, organizational resources, and support for emotion regulation may buffer the risk of exhaustion (Skaalvik and Skaalvik, 2014; Chang et al., 2022; Collie et al., 2012). What therefore needs to be made visible is not any single parent–school conflict, but the sustained psychological tension among emotional rules, external evaluation, and professional judgment.

### 5.3 Boundary work and professional self-protection

Teachers were not fully absorbed by parent-as-client logic. Instead, communicative, pedagogical, evidentiary, and identity boundaries constituted important mechanisms of self-protection. By limiting response time, returning discussions to learning goals, using assessment criteria, explaining curricular logic, and reaffirming their identity as educational professionals, teachers transformed issues that might otherwise be framed as matters of “satisfaction or dissatisfaction” into educational matters open to professional discussion.

Research on boundary management in organizational behavior suggests that individuals need to maintain boundaries when moving across multiple roles in order to reduce role intrusion and psychological depletion (Ashforth et al., 2000). In parent–school communication, boundaries do not signify a refusal to collaborate; rather, they prevent teacher identity from being fully subsumed by the service role. The concept of boundary in educational research also suggests that boundaries may generate tension, but they can also create spaces for negotiation, learning, and the repositioning of relationships (Akkerman and Bakker, 2011). Thus, teachers’ boundary setting does not weaken parent–school collaboration, but shifts collaboration from emotional reassurance and satisfaction-oriented response toward learning goals and professional judgment. Evidence-based communication is a particularly important boundary strategy. By drawing on curriculum objectives, assessment criteria, learning evidence, and written records to explain their judgments, teachers respond to parental expectations while also protecting professional autonomy. Teacher professionalism is not asserted unilaterally; it is continually explained and recognized through interaction (Addi-Raccah and Arviv-Elyashiv, 2008). When teachers are able to distinguish legitimate educational responsibility from excessive service expectations, they may more readily recover a sense of psychological control; perceived autonomy and regulatory space are themselves important resources for sustaining teacher engagement and reducing exhaustion (Skaalvik and Skaalvik, 2014; Pillen et al., 2013).

### 5.4 Tensions and reconstruction of teacher identity

The identity of teachers in marketized international schools is not limited to that of “educator.” They are also expected to be service providers, communicators, representatives of the school brand, providers of emotional reassurance, and professional decision-makers. Multiple identities do not necessarily imply a weakening of professionalism, but they can generate sustained tension among different roles. Teacher identity is dialogic and relational, as individuals form self-understandings among institutional voices, occupational expectations, and personal beliefs (Collie et al., 2012; Akkerman and Meijer, 2011).

This identity tension helps explain why teachers may experience self-doubt and excessive self-monitoring. When parent satisfaction is implicitly incorporated into the evaluation of teachers’ work, dissatisfaction in communication can easily be interpreted by teachers as evidence of their own professional inadequacy. Instability in professional identity may affect teachers’ occupational continuity (Hong, 2010), and identity tension can also have clear emotional consequences and shape teachers’ coping strategies (Pillen et al., 2013). In international schools, this tension arises not only from curricular and cultural transitions, but also from the accumulation of brand commitments, imagined pathways to educational advancement, parental investment expectations, and service logic. Existing research has shown that teachers in Chinese international schools develop agency in intercultural teaching contexts (Lai et al., 2016), and that the wellbeing of international school teachers is also shaped by relational climate and organizational support within schools (Harrison and Kai Hou, 2023). This study adds an emotional dimension that has received comparatively less attention: the reconstruction of teacher identity occurs not only through curriculum, culture, or mobility experiences, but also through the ways teachers manage emotions, set boundaries, and sustain professional judgment. Teacher professionalism in marketized schools has not disappeared; rather, it is redefined between educational judgment and service-oriented response. Professionalism includes not only instructional competence, but also the capacity to explain complex educational decisions, withstand relational pressure, identify the boundaries of responsibility, and maintain ethically grounded educational judgment.

### 5.5 Practical implications

The findings suggest that international schools should translate teachers’ boundary work from an individual coping strategy into explicit organizational arrangements. First, schools should establish clear response-time expectations for parent communication. Routine academic questions may be answered within a defined working-hour window, whereas urgent safety or wellbeing concerns should be directed to a separate emergency channel. This distinction protects teachers’ private time and emotional resources without weakening parent–school collaboration.

Schools should clarify communication hierarchy. Routine learning-related questions may first be handled by the subject teacher or homeroom teacher, while repeated dissatisfaction, cross-subject concerns, or issues involving school policy should be escalated to grade leaders, pastoral coordinators, or senior leadership. Such hierarchy prevents individual teachers from carrying all relational pressure and helps distinguish pedagogical explanation from formal complaint handling.

Teacher protection mechanisms should also be built into parent–school communication. Schools should avoid using parent satisfaction as a direct proxy for teacher quality, provide administrative support for difficult conversations, and recognize evidence-based professional disagreement as part of teaching rather than as service failure. Written templates for curriculum explanation, assessment feedback, and meeting records may help protect professional judgment by making pedagogical decisions transparent.

Professional development should include evidence-based parent communication. Training should help teachers explain curriculum goals, assessment criteria, learning evidence, and follow-up plans, while also practicing emotional regulation and boundary setting. The aim is not to reduce responsiveness to families, but to make responsiveness educationally grounded and psychologically sustainable.

### 5.6 Significance and limitations

This study suggests a pathway of “marketized emotional labor and the negotiation of professional autonomy”: marketized school discourse generates parent-as-client expectations, which in turn shape teachers’ rules of emotional expression; teachers undertake invisible service labor through surface acting, deep acting, and emotional containment; sustained external evaluation places soft pressure on professional autonomy; and teachers maintain psychological resources, professional identity, and occupational wellbeing through boundary work. This pathway extends research on teacher emotional labor from classroom interaction to marketized parent–school relations, and advances research on educational marketization from macro-level institutional analysis to teachers’ micro-level psychological experiences.

This study has several limitations. Insider teacher autoethnography cannot represent all international school teachers. The data were mainly derived from the researcher’s reflections, synthesized critical incidents, and teacher-generated professional materials, and lacked the direct perspectives of parents, students, and administrators. Synthesized narratives enhanced ethical safety, but also reduced the traceability of case-specific details. The study also cannot infer causal relationships among variables. Future research may use multi-case ethnography, interviews, or mixed methods to further examine the relationships among parent-as-client expectations, emotional labor, professional autonomy, and occupational wellbeing, and to compare differences in teachers’ emotional labor and boundary work across different types of schools. Overall, teacher pressure in marketized schools is not merely a matter of individual adaptation, but a psychological process jointly generated by emotional rules, organizational relations, and negotiations of professional identity.

## 6 Conclusion

This study shows that, in marketized Chinese international schools, parent-as-client logic is not an explicit formal system, but an organizational logic embedded in everyday parent–school interaction. Through school branding, service commitments, parent satisfaction, and outcome expectations, it shapes teachers’ rules of emotional expression and extends emotional labor from classroom interaction to parent–school communication. Teachers’ professional autonomy is not simply deprived; rather, it is subjected to soft pressure through anticipatory adjustment, defensive documentation, and adjustment of instructional presentation, and is continuously negotiated. At the same time, teachers are not passive recipients of marketized relations. They maintain professional judgment, psychological resources, and occupational wellbeing through communicative, pedagogical, evidentiary, and identity boundaries. The study further reveals that teacher pressure, emotional exhaustion, identity tension, and professional reconstruction are not isolated experiences, but psychological processes jointly produced within the organizational relations of marketized schooling.

## Data Availability

The original contributions presented in the study are included in the article/Supplementary material, further inquiries can be directed to the corresponding author.
