# Supplementary material for: Boundary work in marketized parent–school relations: an insider autoethnography of teacher emotional labor in a Chinese international school
Source: Front Psychol. 2026 Aug 19;17:1877257. doi: 10.3389/fpsyg.2026.1877257 (PMC13543404; doi:10.3389/fpsyg.2026.1877257)
Supplement: Supplementary file 1 [file Supplementary_file_1.DOCX]

**Appendix 1**. Data Types, Content Descriptions, and Analytic Uses

| **Data type** | **Content description** | **Analytic use** |
| --- | --- | --- |
| Reflective teacher logs | Ongoing records of parent–school communication, pedagogical judgment, emotional regulation, professional autonomy, and identity-related experiences | To capture the everyday processes of teacher emotional labor, pressure on professional autonomy, and boundary management |
| Synthesized critical incident narratives | Reconstructed from recurring situational patterns in the logs and not corresponding to any specific person or event | To represent typical tensions and interaction patterns in marketized parent–school relations |
| Teacher-generated professional materials | Curriculum explanations, homework instructions, assessment criteria, feedback templates, and communication preparation drafts | To examine how teachers explain pedagogical decisions and maintain professional boundaries |
| De-identified institutional discourse materials | Discourses related to individualized education, parent–school collaboration, internationalization, support for educational advancement, and service experience | To understand how marketized school discourse indirectly shapes teachers’ work |
| Reflexive analytic memos | Records of coding, theme development, negative case identification, and reflection on researcher positionality | To trace the interpretive process and reduce single-perspective bias in insider research |
