# Supplementary material for: Boundary work in marketized parent–school relations: an insider autoethnography of teacher emotional labor in a Chinese international school
Source: Front Psychol. 2026 Aug 19;17:1877257. doi: 10.3389/fpsyg.2026.1877257 (PMC13543404; doi:10.3389/fpsyg.2026.1877257)
Supplement: Supplementary file 2 [file Supplementary_file_2.DOCX]

**Appendix 2.** Examples of Synthesized Critical Incident Narratives

The following examples are synthesized and de-identified critical incident narratives developed from recurring patterns in the reflective teacher logs, professional materials, and reflexive analytic memos. They do not refer to any single person, family, class, colleague, school event, or parent–school interaction, but are used to illustrate the structural tensions identified in the analysis.

| **Incident ID** | **Synthesized critical incident** | **Structural tension** | **Teacher strategy** | **Related theme/codes** |
| --- | --- | --- | --- | --- |
| CI01 | The teacher received several non-urgent parent messages in the evening, yet felt that not responding immediately might be interpreted as a lack of care. Instead of replying instantly, the teacher responded during working hours the next day and redirected the complex issue to a formal meeting. | Timely communication was expected, but constant availability weakened emotional and temporal boundaries. | Setting response-time and channel boundaries; transforming immediate emotional pressure into formal explanation. | Client-service logic; emotional reassurance; surface acting; emotional containment; communicative boundary. |
| CI04 | Before releasing a new unit task, the teacher imagined possible parent questions: whether the task was too difficult, whether outcomes would be visible, and whether it was useful for future progression. The teacher rewrote the explanation into objectives, steps, assessment criteria, home support, and FAQs. | Anticipated complaints shaped how instructional decisions were presented. | Frontloading explanation, providing FAQs, clarifying assessment criteria, and preparing written summaries. | Anticipatory adjustment; defensive documentation; instructional presentation adjustment; evidence boundary. |
| CI08 | The teacher gradually built a folder for important instructional decisions, including learning objectives, classroom observations, samples of student work, assessment criteria, and communication summaries. This increased transparency, but also made the teacher feel constantly prepared to be questioned. | Transparency and defensive documentation became intertwined. | Establishing evidence boundaries and using materials rather than emotion to support professional judgment. | Defensive documentation; evidence boundary; self-monitoring; hidden labor. |
| CI09 | After noticing that they repeatedly reread parent messages after work and worried about whether wording might trigger complaints, the teacher added response-time and channel rules to communication notes and distinguished urgent safety issues from general educational discussion. | Care responsibility conflicted with emotional exhaustion. | Resetting response-time rules, clarifying channels, and moving non-urgent matters to working hours. | Boundary work; emotional containment; occupational wellbeing; identity boundary. |
| CI06 | In one negative case, the parent did not demand immediate outcomes but asked how to support classroom goals at home. The teacher provided home observation indicators, and the parent’s feedback helped the teacher revise homework instructions. | Not all parent expectations undermined professional autonomy; collaborative dialogue could support professional work. | Incorporating parent feedback into instructional explanation while retaining curriculum judgment. | Negative case; constructive parent–school collaboration; pedagogical transparency. |
