# Supplementary material for: Boundary work in marketized parent–school relations: an insider autoethnography of teacher emotional labor in a Chinese international school
Source: Front Psychol. 2026 Aug 19;17:1877257. doi: 10.3389/fpsyg.2026.1877257 (PMC13543404; doi:10.3389/fpsyg.2026.1877257)
Supplement: Supplementary file 3 [file Supplementary_file_3.DOCX]

**Appendix 3.** Selected High-frequency Codes, Representative De-identified Excerpts, and Analytic Interpretation

The excerpts below are English translations from the de-identified corpus of reflective logs (RL), professional materials (PM), and synthesized critical incidents (CI). CI entries are synthesized summaries developed from recurring patterns rather than verbatim accounts of single events.

| **Code** | **Frequency** | **Representative de-identified excerpt** | **Short analytic interpretation** |
| --- | --- | --- | --- |
| Anticipatory adjustment | 12 | RL34: “Before releasing a new unit requirement, I first imagined possible misunderstandings. I added FAQs and examples in advance. The preparation felt more like a risk plan.” | Parental expectations shaped how instructional materials were prepared before any direct complaint occurred. |
| Defensive documentation | 20 | PM08: “The folder included curriculum objectives, classroom observation records, homework instructions, student work samples, assessment criteria, meeting summaries, and follow-up action records.” | Professional judgment was turned into a traceable evidence chain, making documentation a form of protective labor. |
| Instructional presentation adjustment | 18 | PM09: “The end-of-unit display will show how students move from questions to sources, organize ideas, and revise their expression. It is not simply an output report, but a visualization of the learning process.” | The instructional goal was not lowered; rather, the learning process was made more visible and understandable to parents. |
| Surface acting | 11 | RL04: “I felt wronged inside, but outwardly kept a patient and positive tone.” | The teacher managed a gap between internal feeling and external expression in order to maintain a stable communicative tone. |
| Deep acting | 3 | RL09: “I tried to understand the parent’s questioning as concern about the child’s frustration rather than as a denial of my teaching.” | The teacher actively reinterpreted the parent’s motive to reduce defensiveness and sustain professional dialogue. |
| Emotional containment | 10 | RL03: “I had to push down my irritation and respond to the parent’s anxiety in a steady tone.” | The teacher absorbed and regulated immediate emotional pressure while keeping the interaction orderly. |
| Professional warmth | 22 | PM14: “Thank you for raising this concern. Based on curricular fairness and students’ long-term development, I cannot provide evaluation content beyond the public scope. I can explain the directions from which the child can prepare.” | Warmth was combined with clear limits, allowing the teacher to respond respectfully without abandoning professional judgment. |
| Communication boundary | 13 | PM06: “General learning issues will be answered during fixed working-hour periods. Urgent safety issues will be prioritized. Complex educational issues should be handled through scheduled meetings or written explanations.” | Boundary setting organized communication by urgency, channel, and timing rather than refusing parent–school contact. |
| Evidence boundary | 33 | RL40: “I used curriculum goals, classroom observations, and the student’s staged performance to explain the reason for the adjustment. With evidence, my tone was steadier; I did not have to seek understanding through emotion.” | Evidence allowed the teacher to support professional judgment through materials rather than through emotional reassurance alone. |
